# Respiratory syncytial virus: Time for surveillance across all ages, with a focus on adults

**DOI:** 10.7189/jogh.14.03008

**Published:** 2024-02-09

**Authors:** Louis Bont, Manuel Krone, Lauriane Harrington, Harish Nair, Terry Nolan, Hitoshi Oshitani, David Salisbury

**Affiliations:** 1 Department of Paediatrics, Wilhelmina Children's Hospital, University Medical Centre Utrecht, Utrecht, the Netherlands; 2 ReSViNET Foundation, Julius Clinical, Zeist, the Netherlands; 3 Infection Control and Antimicrobial Stewardship Unit, University Hospital Würzburg, Würzburg, Germany; 4 Institute for Hygiene and Microbiology, University of Würzburg, Würzburg, Germany; 5 GSK, Wavre, Belgium; 6 Centre for Global Health, Usher Institute, Edinburgh Medical School, University of Edinburgh, Edinburgh, Scotland, UK; 7 MRC/Wits Rural Public Health and Health Transitions Research Unit (Agincourt), School of Public Health, Faculty of Health Sciences, University of the Witwatersrand, Johannesburg, South Africa; 8 Department of Infectious Diseases, The Peter Doherty Institute for Infection and Immunity, University of Melbourne, Melbourne, Victoria, Australia; 9 Murdoch Children’s Research Institute, Melbourne, Victoria, Australia; 10 Department of Virology, Tohoku University Graduate School of Medicine, Sendai, Japan; 11 Programme for Global Health, Royal Institute of International Affairs, Chatham House, London, England, UK

Human respiratory syncytial virus (RSV), a leading cause of serious respiratory illness, can affect individuals of all ages, especially children below two years of age and adults 60 years of age and above, as well as individuals with chronic comorbidities, such as chronic pulmonary or cardiovascular conditions, and immunocompromised individuals [1,2]. In adults, clinical outcomes of RSV infection vary from mild, cold-like symptoms to more serious complications, including pneumonia, exacerbations of chronic medical conditions (e.g. asthma, chronic obstructive pulmonary disease, congestive heart failure), and can lead to death [3]. The RSV-related hospitalisation burden is especially high in older adults. A meta-analysis conducted on data from high-income countries across different continents (based on literature published between 1 January 2000 and 3 November 2021) estimated that approximately 470 000 individuals 60 years of age and above were hospitalised in 2019 due to RSV, of whom approximately 33 000 died. The pooled estimate for RSV acute respiratory infection (ARI) attack rate was 1.62% (95% CI = 0.84–3.08%), corresponding to an estimated 5.2 million RSV-associated ARI cases [2]. As RSV symptoms in adults resemble those of other common respiratory viruses (e.g. influenza), clinical diagnosis of RSV may be challenging.

Circulation of the two major RSV antigenic groups (A and B) is seasonal in temperate climates, with a peak during the winter months, but has a more variable pattern in tropical climates. In addition, RSV circulation overlaps with the influenza season but usually lasts longer (16–22 vs. 6–8 weeks, respectively) [1]. Human respiratory syncytial virus circulation was impacted during the first two years of the coronavirus disease 2019 (COVID-19) pandemic, with RSV cases substantially declining after the widespread implementation of public health and social measures and re-emerging out of season when measures were gradually lifted [4].

Human respiratory syncytial virus surveillance is limited, geographically heterogeneous, and does not systematically include all age groups. While the burden of RSV is highest among very young children, adults 60 years of age and above, and individuals with underlying health conditions, other populations also contribute to RSV transmission. Therefore, improved RSV surveillance systems are needed to better understand the epidemiology of RSV and inform public health measures. To identify the current challenges in RSV surveillance in adults and the ways to expand RSV surveillance systems, an advisory board among seven experts with national and international expertise in infectious diseases and surveillance was held in August 2022. The main points discussed by the group are summarised in plain language in **Figure 1**.

**Figure 1:**
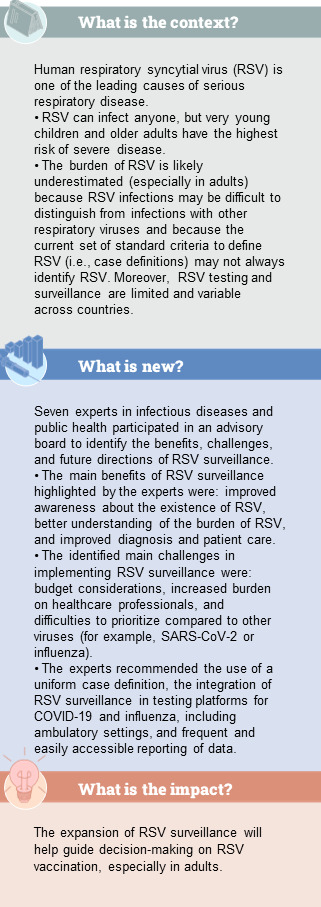
Plain language summary.

## CURRENT SURVEILLANCE SYSTEMS

**Figure 2:**
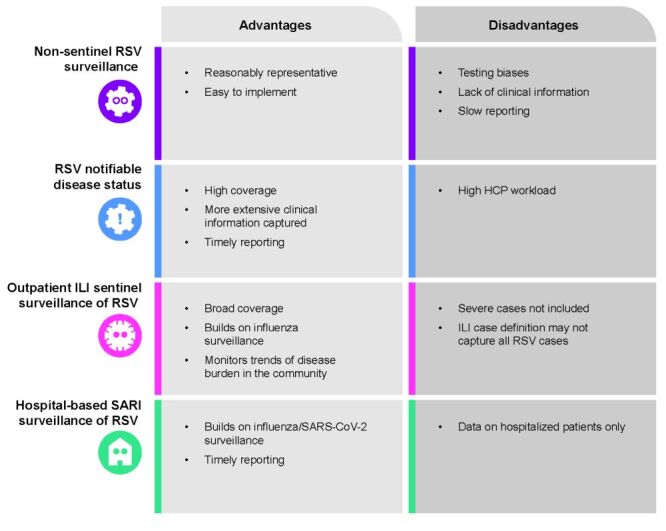
Advantages and limitations of current surveillance systems. RSV – human respiratory syncytial virus, HCP – health care professional, ILI – influenza-like illness, SARI – severe acute respiratory infection, SARS-CoV-2 – severe acute respiratory syndrome coronavirus 2

Human respiratory syncytial virus surveillance systems range from non-sentinel surveillance to notifiable disease status, encompassing different settings (e.g. outpatient, hospital), and with variable inclusion of different ages. The advantages and limitations of each surveillance system were discussed during the advisory board and are summarised in **Figure 2**. In 2017, all countries in the European Union and the European Economic Area, except for Italy, Lithuania, and Luxemburg, either had sentinel or non-sentinel RSV surveillance systems, with many non-sentinel surveillance systems being part of influenza surveillance systems [5]. The World Health Organization (WHO) also integrated RSV surveillance into its Global Influenza Surveillance and Response System, which mostly focuses on children below two years of age [6]. Since 1 September 2022, RSV has become a notifiable disease in Australia, with trends reported in the influenza surveillance report each winter [7]. In the US, the RSV hospitalisation surveillance network, covering about 9% of the US population, collects information on laboratory-confirmed RSV hospitalisations [8].

Several case definitions are used in RSV surveillance, including the ARI case definition, the WHO influenza-like illness (ILI) case definition (i.e. an ARI with measured fever ≥38°C and cough, with onset within the last 10 days), and the severe ARI (SARI) case definition [5,9,10]. However, since the ILI and SARI case definitions include fever, they may not accurately identify RSV [9]. Unlike in children, in whom bronchiolitis is a common clinical presentation of RSV, it may be more challenging to identify RSV in adults as symptoms are variable, depending on age and medical history [1]. In addition, it may be difficult to distinguish RSV from influenza and other non-influenza respiratory viruses considering its non-specific clinical symptoms [9].

## RSV SURVEILLANCE: BARRIERS AND BENEFITS

The group agreed that the benefits of implementing RSV testing and surveillance include a better clinical understanding of RSV, an improved RSV awareness, the ability to provide differential diagnosis and appropriate patient care, the ability to apply outbreak control and transmission mitigation measures, the ability to anticipate hospital resource requirements, and the ability to inform on and measure the public health impact.

However, RSV testing and surveillance may be challenging due to budget considerations, time requirements for health care professionals to perform tests and related tasks, as well as the perceived lack of benefit for patients in the absence of specific treatment and, until recently, of RSV vaccines. The group also highlighted that RSV surveillance systems probably underestimate the burden of disease, especially in older adults who may not be tested or may present at the hospital after the period of testing sensitivity.

The group noted that the main barriers to RSV surveillance were the underrecognised burden of RSV and the lower prioritisation of RSV compared to other infectious diseases (e.g. influenza, COVID-19).

## FUTURE DIRECTION AND RECOMMENDATIONS

The group agreed that a standardised case definition is essential to establish RSV surveillance. To best capture RSV, the group suggested using an ARI case definition instead of the WHO ILI case definition, based on findings that the WHO ILI case definition resulted in an up to 9-fold underestimation of RSV in older adults [10] and in accordance with previously published recommendations [9]. The group also proposed prospective epidemiological studies in both inpatient and outpatient settings to better estimate the burden of RSV in older adults.

The group agreed that RSV surveillance should be integrated within broader respiratory pathogen surveillance and diagnostic systems (e.g. COVID-19 or influenza surveillance). They also noted that testing should include the generation of whole genome sequence data as well as testing in outpatient settings, as not all RSV infections require hospitalisation. The group acknowledged the validity of the testing recommendations shared by Teirlinck and co-authors [9].

The group agreed that children below two years of age and adults 60 years of age and above as well as individuals with underlying health conditions should be included in RSV surveillance efforts. The group also acknowledged that other age groups could be included in RSV surveillance, as they contribute to RSV transmission. Although the group agreed that an efficacious vaccine in adults is important to stimulate the implementation of new surveillance approaches, it also recognised that surveillance may not be needed in all countries for informed decision-making on implementing RSV vaccination if enough data per population are available.

The Preparing for RSV Immunisation and Surveillance in Europe project may provide a good approach to RSV surveillance. The project aims to develop an RSV surveillance platform to help anticipate RSV circulation and outbreaks and to leverage local and national data to understand the impact of RSV burden on health care systems and risk groups. The group considered this international and multi-stakeholder approach as a best practice for consideration across other geographical regions.

The group also noted that data should be readily accessible and frequently reported, preferably in weekly reports integrating multiple ARIs (with, e.g. influenza, RSV, or severe acute respiratory syndrome coronavirus 2). Such reports should include:

– the number of cases

– the number of hospitalisations

– the positivity rate by health care setting

– the timing of infection, to understand seasonality of the disease

– data stratified by test type

– data stratified by age

– data stratified by setting

– data stratified by ascertainment

– data stratified by severity.

## CONCLUSIONS

In conclusion, the expansion of RSV surveillance is important to better understand the epidemiology of RSV in adults as well as to optimise the use of emerging RSV vaccines in this population.
